# Artificial intelligence is transforming disease surveillance, but governance is failing to keep pace

**DOI:** 10.3389/fdgth.2026.1939009

**Published:** 2026-09-09

**Authors:** Abdullahi Osman Mohamed, Abdifatah Afyare, Abdinasir Mohamed Abdi, Yusuf Hassan Muhumed, Mohamed Adam Abdihashi

**Affiliations:** 1 Faculty of Health Science and Tropical Medicine, Somali National University, Mogadishu, Somalia; 2 Faculty of Social Science, Salaam University, Mogadishu, Somalia

**Keywords:** AI governance, artificial intelligence, digital epidemiology, disease surveillance, public health

## Abstract

Artificial intelligence (AI) is transforming disease surveillance by enabling early outbreak detection, predictive modeling, and real-time analysis of diverse health data sources. These advances can strengthen epidemic preparedness and improve public health decision-making. However, AI adoption has progressed faster than the development of effective governance frameworks, creating challenges related to algorithmic bias, transparency, privacy, cybersecurity, accountability, and global equity. Low- and middle-income countries face additional barriers due to limited digital infrastructure and technical capacity. This policy brief highlights the urgent need for responsible AI governance through international standards, explainable algorithms, independent validation, strong data protection, and inclusive stakeholder engagement. Ensuring ethical and equitable AI implementation is essential to maximize its potential for improving disease surveillance and global health security.

To the Editor,

Artificial intelligence is reshaping disease surveillance by enabling earlier outbreak detection, predictive modeling, digital epidemic intelligence, and real-time monitoring from heterogeneous data streams including electronic health records, social media, mobility, environmental, laboratory, and wearable data (1–4). These systems can detect epidemic signals earlier than traditional surveillance, improve spatial and temporal forecasting, and support faster, more targeted public health response, especially when integrated with existing surveillance rather than used as a replacement (5–7).

Yet the governance architecture has not kept pace with this technical acceleration. Recent public health and health AI reviews consistently identify regulatory gaps spanning equity, accountability, privacy, cybersecurity, and lifecycle oversight (5, 8–10). This mismatch matters because disease surveillance operates at population scale, often under conditions of urgency, incomplete data, and asymmetrical power. Algorithmic bias remains a central threat. Biased, incomplete, or nonrepresentative training data can generate unreliable predictions, exclude disadvantaged groups, and intensify existing health inequities (2, 9, 11).

Transparency is an equally important weakness: complex “black box” models remain difficult for public health practitioners to interrogate, while explainable AI methods are still underdeveloped for many surveillance applications (2, 5).

Without explainability, external validation, and traceability, confidence in AI-generated alerts is likely to erode, particularly when models are transferred across settings where epidemiology, infrastructure, and data quality differ (3, 12). Privacy, consent, and data protection are no less consequential. AI surveillance routinely depends on sensitive and often decentralized data, including public digital traces, creating persistent concerns over data ownership, consent, misuse, and security (2, 3, 13).

Cybersecurity and safety vulnerabilities further weaken trust, especially when surveillance outputs can shape high-stakes decisions about risk communication, resource allocation, or movement restrictions (11, 14, 15). Accountability is also poorly specified: when AI-supported surveillance fails, responsibility for error remains blurred across developers, institutions, and public agencies (2, 11). These shortcomings are also geopolitical. Evidence remains concentrated in high-income settings, while low- and middle-income countries often face weaker digital infrastructure, lower local technical capacity, and limited influence over the rules governing AI systems they may eventually import (6, 9, 16). Governance that excludes these settings risks widening inequity while undermining the local validity and legitimacy of surveillance tools. The way forward is not to slow innovation, but to govern it proportionately and internationally. WHO and partner agencies should lead harmonized frameworks for public health AI that require transparency, explainability, independent auditing, external multi-regional validation, and post-deployment performance monitoring across the model lifecycle (3, 6, 9).

These frameworks should embed robust data governance, privacy-preserving approaches, cybersecurity safeguards, and clear human accountability, while ensuring meaningful participation of LMICs, epidemiologists, ethicists, legal scholars, data scientists, and affected communities in oversight and standard setting (2, 11, 15). Artificial intelligence can make disease surveillance faster and smarter, but only responsible governance will make it trustworthy, equitable, and fit for public health.

## Evidence base and literature sources

The evidence presented in this Policy Brief is based on a focused narrative review of the contemporary literature rather than a formal systematic review. Relevant publications were identified through targeted searches of PubMed, Scopus, and Web of Science, together with recent guidance and policy documents published by the World Health Organization (WHO) and other international organizations. Priority was given to peer-reviewed primary studies, systematic reviews, and authoritative policy documents published between 2023 and 2026 addressing artificial intelligence, infectious disease surveillance, AI governance, ethics, transparency, accountability, and digital health regulation. References were selected according to their direct relevance to AI governance in public health surveillance. As a narrative review, this article may not capture all available evidence; therefore, the recommendations should be interpreted as evidence-informed policy perspectives based on the current literature.

## Recommendations

AI-enabled disease surveillance should be governed through internationally harmonized frameworks that mandate transparency, explainability, independent algorithm auditing, external validation, robust data governance, cybersecurity safeguards, and continuous post-deployment monitoring. Capacity building in low- and middle-income countries, alongside multidisciplinary oversight involving public health experts, ethicists, legal scholars, and affected communities, is essential to ensure equitable, trustworthy, and accountable implementation.

In conclusion, artificial intelligence has the potential to revolutionize disease surveillance by improving the speed, accuracy, and effectiveness of outbreak detection and response. However, without robust governance, these benefits may be undermined by ethical, legal, and equity challenges that erode public trust. Advancing responsible AI governance through transparent, accountable, and globally inclusive frameworks is therefore essential to ensure that AI strengthens, rather than compromises, public health surveillance.

## References

[1] Kraemer MUG Tsui JLH Chang SY Lytras S Khurana MP Vanderslott S. Artificial intelligence for modelling infectious disease epidemics. Nature. (2025) 638:623–35. 10.1038/S41586-024-08564-W 39972226 PMC11987553

[2] Villanueva-Miranda I Xiao G Xie Y. Artificial intelligence in early warning systems for infectious disease surveillance: a systematic review. Front Public Health. (2025):13:1609615. 10.3389/FPUBH.2025.1609615 40626156 PMC12230060

[3] Li JH Tseng YJ Chen SH Chen KF. Artificial intelligence in infection surveillance: data integration, applications and future directions. Biomed J. (2026) 49:100929. 10.1016/J.BJ.2025.100929 41205676 PMC13091077

[4] Mayaki LD. Enhancing real-time infectious disease surveillance through AI-driven early warning and predictive outbreak detection systems. GSC Biol Pharm Sci. (2025) 33:001–14. 10.30574/GSCBPS.2025.33.3.0484

[5] MacIntyre CR Chen X Kunasekaran M Quigley A Lim S Stone H. Artificial intelligence in public health: the potential of epidemic early warning systems. J Int Med Res. (2023) 51:3000605231159335. 10.1177/03000605231159335 36967669 PMC10052500

[6] Mendes VIS Mendes BMF Moura RP Lourenço IM Oliveira MFA Ng KL. Harnessing artificial intelligence for enhanced public health surveillance: a narrative review. Front Public Health. (2025) 13:1601151. 10.3389/FPUBH.2025.1601151 40809756 PMC12343694

[7] Olaboye JA Maha CC Kolawole TO Abdul S. Innovations in real-time infectious disease surveillance using AI and mobile data. Int Med Sci Res J. (2024) 4:647–67. 10.51594/IMSRJ.V4I6.1190

[8] Goktas P Grzybowski A. Shaping the future of healthcare: ethical clinical challenges and pathways to trustworthy AI. J Clin Med. (2025):14:1605. 10.3390/JCM14051605 40095575 PMC11900311

[9] Gao Q Chen L Huang Z. Opportunities and challenges of artificial intelligence in public health: a systematic review on technological efficacy, ethical dilemmas, and governance pathways. Front Public Health. (2026):13:1748797. 10.3389/FPUBH.2025.1748797 41613098 PMC12847321

[10] Al Kuwaiti A Nazer K Al-Reedy A Al-Shehri S Al-Muhanna A Subbarayalu AV. A review of the role of artificial intelligence in healthcare. J Pers Med. (2023) 13:951. 10.3390/JPM13060951 37373940 PMC10301994

[11] Zhang J Zhang Z-M. Ethics and governance of trustworthy medical artificial intelligence. BMC Med Inform Decis Mak. (2023) 23:7. 10.1186/S12911-023-02103-9 36639799 PMC9840286

[12] Silcox C Zimlichmann E Huber K Rowen N Saunders R McClellan M. The potential for artificial intelligence to transform healthcare: perspectives from international health leaders. NPJ Digit Med. (2024) 7:88. 10.1038/S41746-024-01097-6 38594477 PMC11004157

[13] Radanliev P. Privacy, ethics, transparency, and accountability in AI systems for wearable devices. Front Digit Health. (2025) 7:1431246. 10.3389/FDGTH.2025.1431246 40599875 PMC12209263

[14] Panteli D Adib K Buttigieg S Goiana-da-Silva F Ladewig K Azzopardi-Muscat N. Artificial intelligence in public health: promises, challenges, and an agenda for policy makers and public health institutions. Lancet Public Health. (2025) 10:e428–32. 10.1016/S2468-2667(25)00036-2 40031938 PMC12040707

[15] Borham A Kamal LT Chun S. Artificial intelligence in epidemic watch: revolutionizing infectious diseases surveillance. Front Digit Health. (2025) 7:1692617. 10.3389/FDGTH.2025.1692617 41425282 PMC12711821

[16] Tripathi DA. Artificial intelligence in public health surveillance: a cross-disciplinary assessment of predictive analytics and ethical concerns. Edulogic Int J Multidiscip Res. (2025) 01:13–26. 10.63665/EIJMR.V01I01.2

